# Thermal Studies of Fractionated Lignite and Brown Coal Fly Ashes

**DOI:** 10.3390/ma17143464

**Published:** 2024-07-12

**Authors:** Jurij Delihowski, Marcin Gajek, Piotr Izak, Marcin Jarosz

**Affiliations:** 1 Faculty of Materials Science and Ceramics, AGH University of Science and Technology, 30-059 Krakow, Poland; 2 Comex Polska Sp. z o.o., 30-644 Krakow, Poland

**Keywords:** coal fly ash, grain size fractions, thermal behaviour, high-temperature treatment, thermal reactivity

## Abstract

Coal fly ash (CFA), a by-product of coal combustion, is a valuable raw material for various applications. However, the heterogeneous nature of the composition and properties of CFA provides challenges to its effective usage and utilisation. This study investigates the thermal behaviour of the fly ashes of lignite (FA1) and brown coal (FA2) and their fractions obtained by dry aerodynamic separation. Thermal analysis techniques, including thermogravimetry (TG), differential scanning calorimetry (DSC), and evolved gas analysis (EGA), were used to characterise the behaviour of the fly ash fractions while heating up to 1250 °C. The results reveal distinct differences in the thermal behaviour between ash types and among their different size fractions. For the FA1 ashes, the concentration of calcium-rich compounds and the level of recrystallisation at 950 °C increased with the decrease in particle size. The most abundant detected newly formed minerals were anhydrite, gehlenite, and anorthite, while coarser fractions were rich in quartz and mullite. For the FA2 ashes, the temperature of the onset of melting and agglomeration decreased with decreasing particle size and was already observed at 995 °C. Coarser fractions mostly remain unchanged, with a slight increase in quartz, mullite, and hematite content. Recrystallisation takes place in less extension compared to the FA1 ashes. The findings demonstrate that the aerodynamic separation of fly ashes into different size fractions can produce materials with varied thermal properties and reactivity, which can be used for specific applications. This study highlights the importance of thermal analysis in characterising fly ash properties and understanding their potential for utilisation in various applications involving thermal treatment or exposure to high-temperature conditions. Further research on advanced separation techniques and the in-depth characterisation of fly ash fractions is necessary to obtain materials with desired thermal properties and identify their most beneficial applications.

## 1. Introduction

Coal fly ash (CFA) is a by-product of coal combustion in power plants, consisting of fine particles of various minerals, unburned carbon, and other impurities [1,2,3]. The global production of coal ash exceeds 750 million tonnes annually, highlighting the great potential for the repurposing of this industrial waste material [4,5,6]. CFA has been widely used as a substitute for cement materials [1,4,6], a filler in concrete and asphalt [7,8,9,10], a soil stabiliser [11,12,13,14], and as a geopolymer precursor [15,16,17], and as an additive for ceramics and building materials [18,19,20]. However, the effective utilisation of CFA is often limited by its heterogeneous composition and properties, which depend on factors such as the source and type of the parent coal, combustion conditions, and post-combustion treatments [21,22,23,24].

Some production lines include high-temperature thermal treatment. As a result, a complex series of transformations takes place across different temperature ranges. These changes include dehydration, the combustion of organic matter, mineral decomposition, and phase transitions [25,26,27,28,29]. The heterogeneous nature of fly ash can lead to unexpected behaviour during thermal processing, potentially limiting its use in high-temperature applications.

As was reported by Kim et al. [30], the residual coal in ashes is undesirable in the production of ceramic tiles with ash additives. During the sintering of tiles, the carbon residuals of the fly ashes undergo oxidation with the release of CO_2_ gas. Therefore, it is important to hold the tiles at a temperature that is sufficient for carbon oxidation before the pores supplying oxygen to the inside of the ceramic tile are sealed.

From another perspective, during the production of lightweight aggregate, it is desirable to envelope the gases emitted within the aggregate, giving as a result the expansive foaming of the material [31,32]. In this case, the increase in the amount of organic residuals in the ash may be desirable as a foaming agent, while the mineral part of the ashes would build up the aggregate body [33]. The retention of bubbles inside the aggregate is possible due to the formation of the viscous liquid phase prior to the gas-forming reactions. Knowledge of the temperature of the appearance of the melted viscous phase and the gas emission temperatures (both from organic oxidation and other volatile compounds) is important to obtain the desired quality of the final product [33,34,35,36,37,38].

However, during cement clinker production, fly ash is introduced primarily as a supplementary cementitious material and a raw material substitute [39,40,41,42]. The high silica content of fly ash enhances the formation of pozzolanic and hydraulic phases in the clinker, improving the long-term strength and durability of the final cement products [42,43,44]. Desired properties include a high alumosilicate glass content, a low carbon content, and appropriate particle fineness and ensure efficient clinker formation and high-quality cement [45,46].

The incorporation of fly ash in geopolymer synthesis offers a sustainable approach to construction materials by utilising industrial by-products as reactive aluminosilicate sources. The efficacy of fly ash in geopolymers is based on its physicochemical properties, particularly its amorphous silica and alumina content, particle size distribution, and glass content [15,47,48] These characteristics directly influence the geopolymerisation process and the properties of the resultant material. The thermal stability of fly ash-based geopolymers improves with increasing fly ash content, as demonstrated by the thermogravimetric analysis by Catauro et al. [49] However, variations in the composition of fly ash can significantly affect the performance of the geopolymer. Insufficient reactive components or excess carbon content may lead to incomplete geopolymerisation or interfere with alkaline activation [50]. Optimal alkaline activator concentration and curing conditions must be tailored to specific fly ash properties to ensure the desired mechanical and durability results [16,51]. Thus, careful quality control and mixing design optimisation are critical to harnessing the full potential of fly ash in geopolymer production.

The fitting of the process cycle in the condition of constantly changing properties, and thus the quality of fly ashes, can be complicated and exclude the potential uses. As a result, several technological solutions were developed and used to obtain the desired properties of CFA and improve its suitability for various applications [25,26,27,28,29]. Those methods include several separation techniques [52,53,54,55,56], and mechanical [57,58,59], chemical [60,61,62,63], and thermal activation [26,27,64,65,66,67]. 

On the contrary, when separated into different fractions, each having individual stable properties, fractionated ashes may exhibit more consistent and predictable characteristics, expanding their potential for targeted applications [21,52,68,69,70,71].

This study investigates the thermal behaviour of two different types of coal fly ash (CFA): lignite-derived (FA1) and brown coal-derived (FA2), with a particular focus on the influence of particle size distribution. The CFA samples, obtained through a dry aerodynamic classification process, were subjected to comprehensive thermal analysis using thermogravimetry (TG), differential scanning calorimetry (DSC), and evolved gas analysis (EGA) techniques. The results of the thermal analysis were correlated with the mineralogical and chemical composition of the ashes to provide a comprehensive understanding of their thermal properties and reactivity.

The primary objective of this research is to identify and explain the thermal transformation processes and reactivity patterns of two distinct Polish CFAs under high-temperature conditions. The characterisation of these ashes across different size fractions seeks to reveal their potential to produce high-quality, application-specific materials, thus expanding their utilisation in various industrial applications. This study contributes to the larger goal of optimising CFA usage, potentially leading to more sustainable practices and reduced environmental impact. The findings presented here not only advance our understanding of CFA thermal properties but also provide a foundation for developing innovative, tailored solutions for CFA utilisation in high-temperature applications. This research represents a significant step toward more efficient resource management and the development of environmentally friendly materials in the context of circular economy principles.

## 2. Materials and Methods

The fly ashes from two Polish coal-fired power plants were studied. The first fly ash (FA1) was obtained from the Belchatow power plant from lignite combustion, and the second fly ash (FA2) was obtained from the Krakow power plant from bituminous coal.

**Figure 1:**
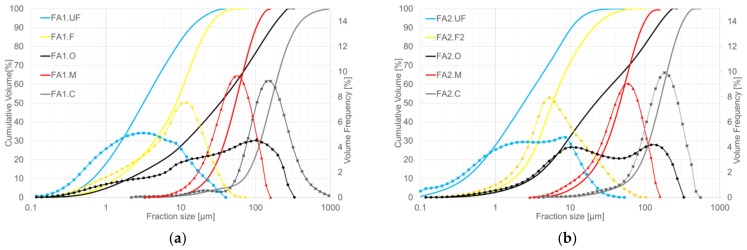
Granulometric distribution of the fly ash fractions obtained: (**a**) FA1 ashes; (**b**) FA2 ashes.

The dry aerodynamic fractionation of raw ashes was performed on ACX aerodynamic classifiers provided by Comex Polska sp. z o.o. [72]. ACX classifiers possess the capability to fractionate materials with exceptional precision, achieving particle separation of up to 2–3 microns (d97). The nature of the separation process is based on the behaviour of the particles in the airstream while exposed to the radial centrifugal forces created by the impeller with rotational blades. The separation process is generally regulated by the airstream flow speed and the rotational speed of the impeller. Representative fractions were obtained and divided into the following groups: O, original ash; C, coarse fractions; M, middle fractions; F, fine fractions; UF, ultrafine fractions. The granulometric distribution is presented in Figure 1.

The chemical composition of the ashes was determined using the Bruker WD-XRF S8 TIGER spectrometer. The measurements were made using the vacuum method with the built-in Quant Express reference standard. The results are presented in Table 1.

To investigate the mineral changes under the influence of thermal treatment, the samples were placed in a furnace and heated in a step schedule for 2 h to 950 °C, where after roasting for 1 h at maximum temperature the samples were cooled to room temperature in a desiccator. The samples obtained were named as follows: O.t, original ash; C.t, coarse fractions; M.t, middle fractions; F.t, fine fractions; UF.t, ultrafine fractions for both FA1 and FA2, respectively, and an XRD analysis was performed.

**Figure 2:**
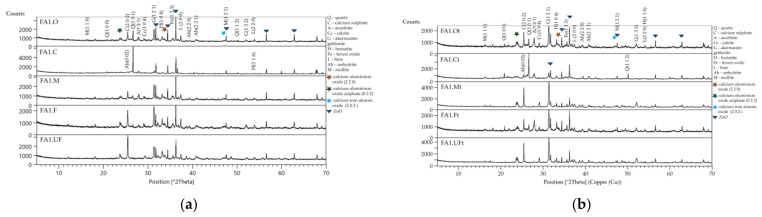
XRD results of FA1 fractions: (**a**) untreated; (**b**) treated at the 950 °C fraction.

**Figure 3:**
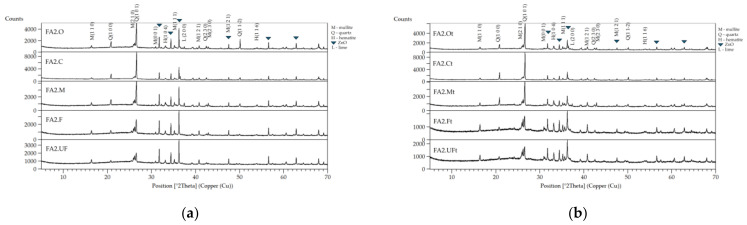
XRD results of FA2 fractions: (**a**) untreated; (**b**) treated at 950 °C fraction.

The PANalytical Empyrean X-ray diffractometer was used for the phase composition determination. The measurements were made with the following settings: monochromatic radiation with a wavelength corresponding to the copper K(α1) emission line (1.54178 Å), the angular range of 5–90 degrees in 2θ scale, with a goniometer step size of 0.008°. The X’Pert HighScore Plus computer software by PANalytical was used for phase composition qualitative analysis utilizing the PDF-2 database and the ICSD database. The mineralogy composition of the raw and thermally treated ashes of FA1 and FA2 is presented in Table 2 and Table 3, and Figure 2 and Figure 3.

Scanning electron microscopy (SEM) images were obtained for both the raw fly ashes and thermally treated ashes by using the Thermoscientific Fisher Phenom XL equipment.

**Figure 4:**
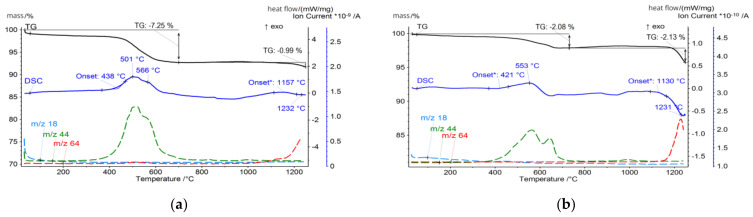
Thermal analysis of (**a**) FA1.C fraction; (**b**) FA1.M fraction.

**Figure 5:**
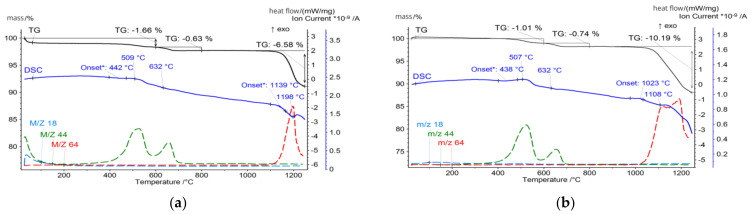
Thermal analysis of (**a**) FA1.F fraction; (**b**) FA1.UF fraction.

**Figure 6:**
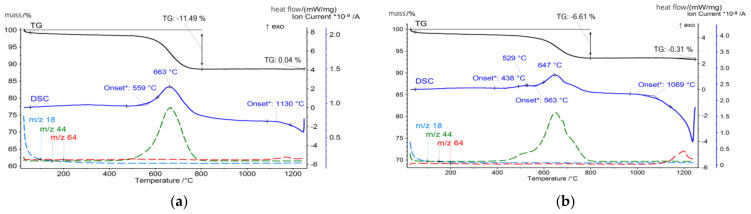
Thermal analysis of (**a**) FA2.C fraction; (**b**) FA2.M fraction.

**Figure 7:**
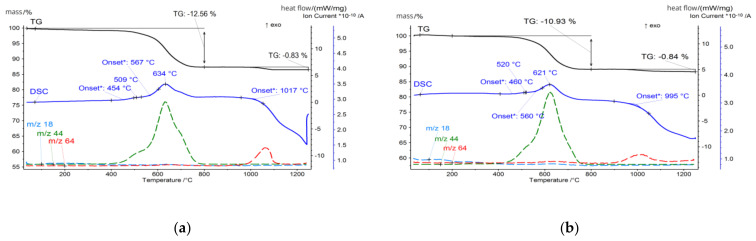
Thermal analysis of (**a**) FA2.F fraction; (**b**) FA2.UF fraction.

The CFA samples were subjected to a thermal analysis that included thermogravimetry (TG), differential scanning calorimetry (DSC), and evolved gas analysis (EGA) techniques, and the results were correlated with the mineralogical and chemical composition of the ashes. For thermal analysis measurements with the detection of gas emission, the STA 449 F3 Jupiter (Netzsch) coupled to the QMS Aëolos quadrupole mass spectrometer was used. The measurements were made in platinum crucibles in synthetic air and argon atmosphere (flow 20 mL/min, each). The samples were heated from 30 °C to 1250 °C at a rate of 10 °C/min. The influence of heating rate on the thermal events was not taken into account, but it is known that slower heating rates would improve the resolution of thermal events and ensure uniform temperature distribution, but it will significantly increase analysis time. To optimise the measurement process, the moderate heat rate was chosen, offering a good balance between capturing detailed thermal events and maintaining a practical analysis duration. However, the influence of the heating rate on thermal events might be the target of further investigations. The temperature-related curves obtained are presented in Figure 4, Figure 5, Figure 6 and Figure 7. The total loss of ignition (LOI) was obtained according to the ASTM D7348 procedure and is presented in Table 4.

## 3. Results and Discussion

The thermal behaviour of the calceus FA1 and silicious FA2 coal fly ashes was investigated using thermogravimetric analysis (TGA), differential scanning calorimetry (DSC), and gas emission analysis. The obtained results allow us to distinguish certain temperature regimes accompanied by specific processes and transformations, which are discussed below.

Up to 300 °C, for both the FA1 and FA2 ash fractions, a gradual decrease in the TG curve related to the evaporation of physically bound water (*m*/*z* 18) was observed. Most of the water was absorbed by the fly ash particles from atmospheric moisture during storage because of its hygroscopic properties. For the tested samples, the maximum mass loss in this area was obtained for the coarse FA1.C and FA2.C fractions states 1.2% and 1.3%, respectively, while for the finer fractions FA1.UF together with FA2.F and FA2.UF the water release was detected in marginal amounts, suggesting that the ashes were stored and processed in dry conditions [73,74,75,76]. Probably the detected water is residual water that originates from the parent coal, and that remains in the volume of the ash particles, concentrating in the coarser grains. Notably, the waterless processing of fly ashes offers significant advantages, as it prevents any contact with water. Preserving all the beneficial properties offers a wide range of potential utilisation pathways, which will be discussed later.

Water emission at higher temperatures, the 400–800 °C region, is almost negligible for both ashes. However, according to studies [26,29,77], the presence of Ca(OH)_2_ or hydrated calcium silicates in fly ashes can contribute to water emission in this temperature range due to the endothermal dihydroxylation reaction. In this study, the mineralogical results obtained by XRD did not detect the presence of Ca(OH)_2_ in the samples tested, but some of the complex calcium silicates may have hydrated forms as described by [78], which can be a source of marginal water trace.

However, the thermal behaviour of tested samples becomes dominated by those reactions in the 400–800 °C temperature region. For FA1, the onset of this reaction for the FA1.UF and FA1.C fractions takes place at 438 °C and reaches its intensity at 507 °C and 501 °C, while obtained by the TG measurements the mass loss in the 400–700 °C region is around 1.5% and 7.2%, respectively. Meanwhile, for brown coal ashes FA2, the organic combustion reaction has its onset at 560 °C, and for the FA2.UF and FA2.C fractions reach peak intensity at 621 °C and 663 °C, which is higher compared to the FA1 ashes, while mass loss calculated in the region of 450–780 °C states around 10.9% and 11.5%, respectively.

**Figure 8:**
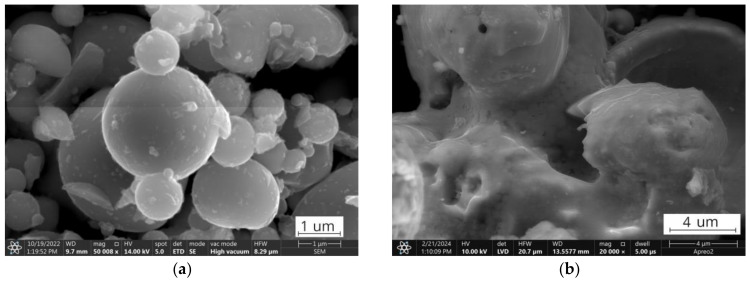
SEM images. (**a**) FA2.UF fraction; (**b**) FA2.UFt fraction.

However, the difference in the combustion temperature regions for the FA1 and FA2 ashes, and within fractions, suggests the presence of various types of residual organics with varying structural organizations [79,80,81]. The relative amounts of each representation depend on the type of coal, the combustion conditions, and the post-combustion processes [82,83,84]. Instantly, low-rank lignite coals have a higher burnout and coarser unburned carbon distribution, while high-rank bituminous coals have a lower level of burning efficiency and are more prone to soot formation [85,86,87,88]. As a result, the FA1 ashes have an overall lower level of mass loss due to coal residues compared to the FA2 ashes. Obtained from higher-ranked coal, the FA2 ashes overall show higher ignition and combustion temperatures compared to the low-ranked FA1 coal ashes [80,81,89]. Furthermore, the granulometric classification performed concentrates fine organic particles, soot, and coal grain fragments in finer fractions, while large uncombusted grains remain in coarser fractions, resulting in a reduction in the peak temperature of combustion processes for the finer compared to the coarser fractions [70,88,90]. In Figure 8a, soot can be observed as two different types. The first type is individual spherical particles with diameters much smaller than those of the aluminosilicate fly ash particles at around 10–50 nm. The second type can be observed as nanometric agglomerates, appearing as individual clusters on the surfaces of larger grains [85,86,88]. However, other mineral residuals, such as aluminosilicate glass or volatile condensed matter, can form similar formation types and from SEM images it could be hardly distinguished due to sub-nanometric size [81,89,91,92,93]. Organic petrographical studies could be performed in order to obtain direct information about the quantitative and qualitative composition of organic fly ash, as was performed in the study [94]. Soot, composed of fine carbon particles and organic compounds formed during the incomplete combustion of carbonaceous materials through pyrolysis, nucleation, growth, and agglomeration, and due to its high surface area, reactive functional groups, catalytic presence, porosity, and amorphous structure, contributes to the lowering of combustion temperature during the thermal treatment of fly ashes [25,86]. It contributes to the overall decrease in combustion temperature for finer fractions. Figure 8b presents the FA2.UF fraction after the 950 °C treatment, where these nanoscale grains and clusters do not appear, indicating their thermal decomposition, regardless of their composition and origin.

**Figure 9:**
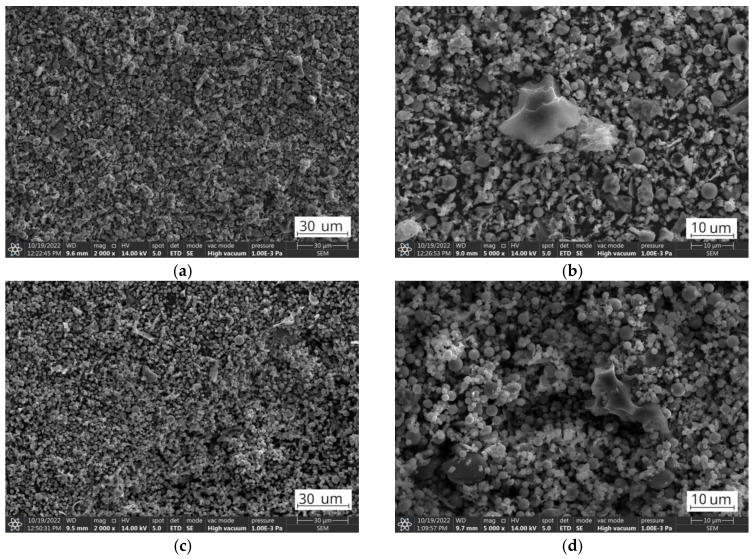
SEM images of ultrafine fractions: (**a**,**b**) FA1.UF fraction; (**c**,**d**) FA2.UF fraction.

However, from the analysis of the TG mass loss and CO_2_ gas emission curves, organic residuals from the FA1 ashes are concentrated mainly in the coarse fraction, while the FA2 ashes have a higher level of fine-grained organics compared to the FA1 ashes. For the FA2 ashes, only the middle fraction shows a relative decrease in organic residuals indicating the distribution of organics in fine and coarse fractions and its lack in middle-size fractions (Table 4). In addition, the authors suggest that for the FA1 ashes, at least two types of residual coal can be distinguished. This can be observed as two individual reactions of CO_2_ gas emissions (*m*/*z* 44) reaching their maximum at 507 °C and 632 °C for the fraction FA1.UF fraction (Figure 5). For the FA2 ashes, the combustion takes place as one continuous reaction with only slight deviations of CO_2_ emissions, suggesting a more similar carbon content. However, the CO emissions deviations of the FA2 ashes could be better observed at the FA2.M fraction. Probably due to the overall lower concentration of organic residuals in this middle fraction, compared to coarse and fine ones, the total CO_2_ emission curve is more sensitive to disturbances from secondary minor reactions. Therefore, the deviation caused by various types of minor reactions can be observed at 509 °C and 643 °C on the CO_2_ emission curve for the FA2.M fraction, indicating a variety of carbon-containing forms in the FA2 ashes [79,82,83,85,86]. Examples of the untreated ultrafine fractions are presented in Figure 9 and some of the residual coal grains can be observed as coarser particles surrounded by finer nonorganic residuals. It should be noted that for FA2.UF, spherical-shaped grains have a dominant share in the constitution being easy-to-melt aluminosilicate microspheres, while FA1.UF is presented as a combination of spherical grains and irregularly shaped Ca-bearing particles, which will also influence further thermal behaviour and will be discussed later.

Some of the other minor reactions related to CO_2_ emission for both ashes could be the decarbonation of calcium carbonate (CaCO_3_) and other calcium-containing minerals taking place at 700–800 °C [22,26,29,95]. As reported by [96,97,98,99,100], the intensity and exact position of this peak can vary depending on the crystallinity, particle size, and composition of the carbonate phases and can occur at wide temperatures, ranging from 600 to 900 °C. In the current study, the mineralogical results did not detect calcium carbonate phases, suggesting its marginal amount below the detection threshold or its complete absence.

**Figure 10:**
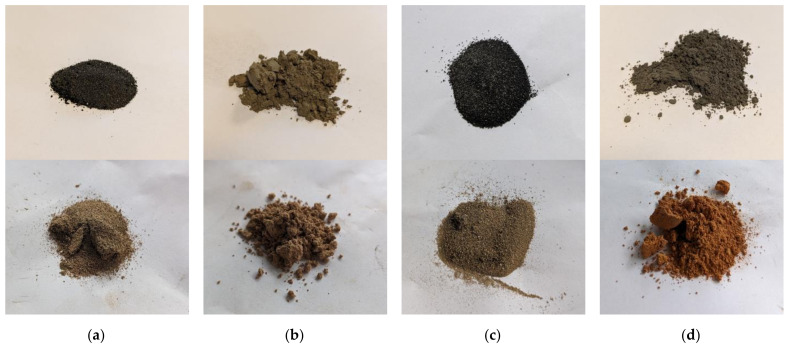
Photo image of fly ashes before (**top**) and after thermal treatment (**bottom**) at 950 °C for 1 h: (**a**) FA1.C; (**b**) FA1.UF; (**c**) FA2.C; (**d**) FA2.UF.

Some researchers relate reactions in the region up to 670 °C to the oxidation and devitrification of Fe-bearing compounds included in fly ash [29,89]. According to studies [25], magnetite-to-hematite transitions can occur in two peaks at 375 °C and 580 °C, where the first occurs at 375 °C, with oxidation occurring on the surface, and the second occurs in the bulk. Oxidation during thermal treatment can be observed as the colour change in fly ash samples from blackish-grey to russet (Figure 10). As can be concluded from the XRD analysis (Table 2 and Table 3), the increase in hematite content was observed to the greatest extent for the FA1.M and FA2.M fractions, resulting in a gain of 3.6% and 2.6% in raw ashes to 12.3% and 10.1%, respectively, after the treatment at 950 °C.

The coarse fractions of both types of ash exhibit a notably darker colouration compared to their fine counterparts, indicative of a higher concentration of carbonaceous particles. This colour gradient across the fractions undergoes a significant transformation during thermal treatment, transitioning from dark to a lighter, sand-like hue. This chromatic evolution is attributed to the combustion of residual carbon and the oxidation of iron-bearing compounds as was mentioned earlier. The colour characteristics of the raw ash fractions and their alteration during thermal processing can have implications for certain specialised applications in the construction industry where aesthetic considerations are paramount [101,102,103]. In scenarios where the inherent colouration is deemed unsuitable, additional thermal treatment targeting the combustion of organic content or the oxidation of iron-bearing compounds may be employed to achieve the desired chromatic properties. Alternatively, electrostatic or electromagnetic separation techniques can be utilised to selectively remove specific components, yielding distinct product streams such as uncombusted coal concentrate, iron-rich fraction, and a purified, carbon- and iron-depleted ash fraction. These separation methodologies not only allow colour modification but also enhance the overall versatility and applicability of ash fractions in various industrial contexts [54,55,84,104,105].

The 800–950 °C region: after the completion of the aforementioned reactions, both FA1 and FA2 ashes show overall thermal stability, with minimal reactions.

**Figure 11:**
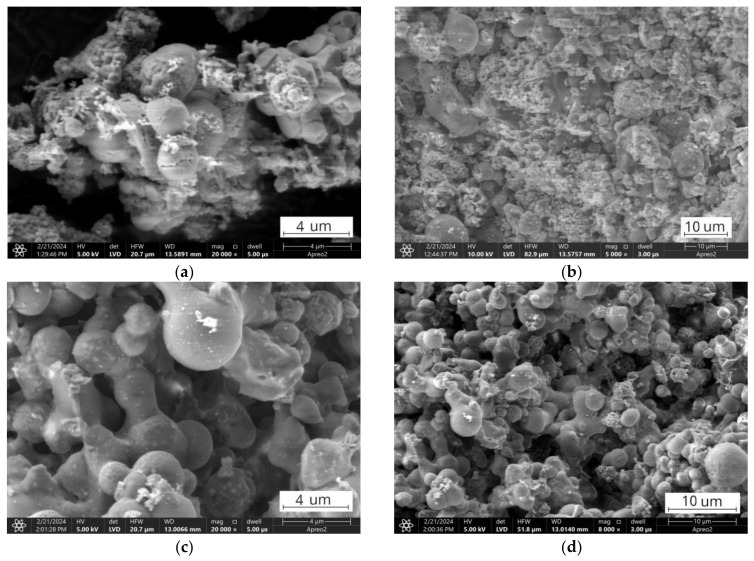
SEM images of ultrafine fractions after thermal treatment at 950 °C: (**a**,**b**) FA1.UFt fraction; (**c**,**d**) FA2.UFt fraction.

Above 950 °C, a continuous, mostly endothermal reaction can be observed for both ashes and is more prominent for the finer fractions. The behaviour of the thermal curves in this region can be shaped by the complex interactions between various minerals in the ashes and includes the formation, decomposition, and modification of different phases [25,26,29,106]. One of those reactions is the melting of aluminosilicate glasses, which can start even below 950 °C [29,106,107,108]. The thermoplastic behaviour of the tested fractions is significantly influenced by both the rank of the parent coal and the size of the fraction size [109,110]. High-rank coals typically contain a higher proportion of silica (SiO_2_) and alumina (Al_2_O_3_) compounds compared to low-rank coals [22,73,91,111]. These glassy phases and aluminosilicate minerals in the ashes soften and melt at elevated temperatures, resulting in a more pronounced thermoplastic behaviour (Figure 11c,d) [107,110]. On the contrary, low-rank coal ashes exhibit a lower tendency toward thermoplasticity, which is related to the presence of a calcium-rich mineral phase, including calcium oxide (CaO), calcium sulphate (CaSO4), and calcium aluminosilicates (Figure 11a,b) [112,113,114,115].

**Figure 12:**
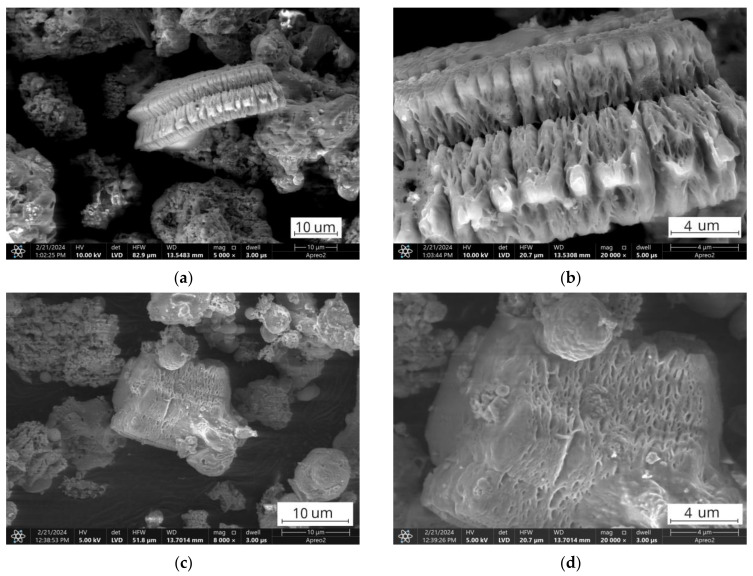
SEM images of the FA1.Mt fraction showing unmelted Ca-rich minerals: (**a**,**c**) Overview of particle morphology; (**b**,**d**) Close-up view.

**Figure 13:**
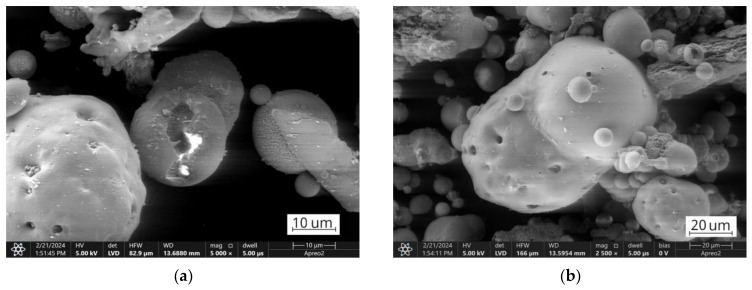

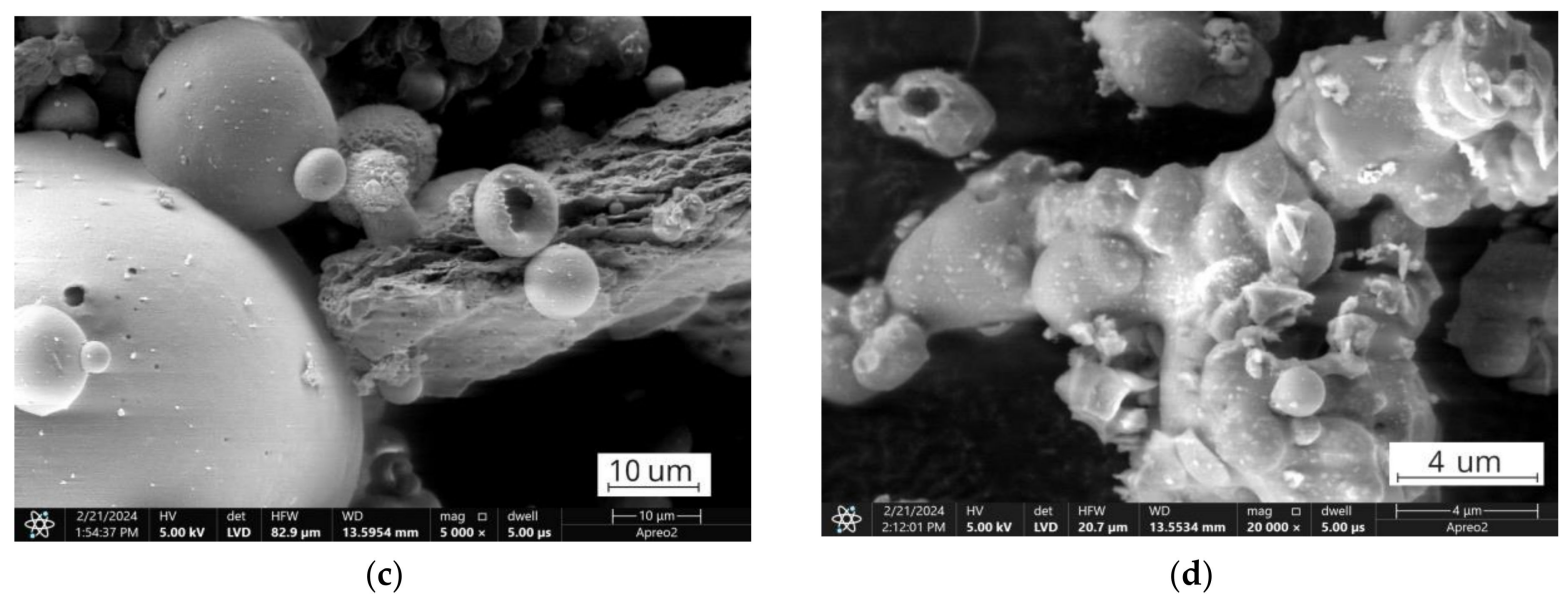
SEM images of the FA2.Mt fraction showing fused grains: (**a**) Overview of fused particle morphology; (**b**) Close-up of smooth, rounded fused particles; (**c**) Necking and bridging between fused particles; (**d**) Agglomerated fused particles forming larger structures.

These calcium-rich minerals can have higher melting points compared to aluminosilicate glass, increasing the thermal stability of the calcareous ashes [107,109,113]. In the FA1 ashes, the presence of crystalline minerals such as anhydrite, anorthite, and other mineral phases can act as physical barriers between easy-to-melt glassy particles, preventing their fluxing and sintering (Figure 12) [26,29,106,116,117,118,119]. On the contrary, the FA2 ashes had a higher proportion of silica and alumina glass compounds with lower melting points that undergo flux and sintering, creating necks and bridges between, leading to the observed thermoplastic behaviour and agglomeration in the FA2 ashes tested at 950 °C (Figure 13) [118,119,120,121,122]. For the finer fractions, these reactions occurred at slightly lower temperatures than for the coarser ones, suggesting the influence of particle fineness and the concentration of specific mineral phases. Furthermore, the decrease in fraction fineness results in a decrease in the onset fluxing temperature due to the presence of higher levels of alkaline modifiers in the glass content in the finer fractions that act as fluxing agents due to depolymerisation of the glass structure [91,123,124,125,126,127,128], and the results of the distribution of chemical composition confirm this tendency (Table 1).

As the molten phase appears, it can participate in the partial dissolution of other minerals and compounds [121,122]. These dissolutions can react with other compounds to form new phases or to reprecipitate, contributing to the growth of existing minerals and in formation of new crystal forms. For example, as reported by Nguyen et al. [129], mullite contaminations in fly ash undergo decomposition into silica and alumina at temperatures above 1600 °C, which, together with magnesium oxide, can form spinel. The appearance of the spinel was detected in the fine and ultrafine fractions of FA2 after the thermal treatment at 950 °C, suggesting that other compounds (such as alumosilicate glass) could be the source of silica and alumina to form the spinel. Furthermore, recrystallisation can also occur through the rearrangement and diffusion of ions within the solid phase, and the FA1 and FA2 ashes contain some amorphous phases that undergo devitrification during thermal treatment [26,106,121,130].

For instance, the content of gehlenite-akermatine (Ca_2_Al_2_SiO_7_) increases significantly, indicating the devitrification of the amorphous phase and crystal growth. The most evident of such reactions can be observed in the fine FA1.F and FA1.UF fractions, where the gehlenite content increased from 17.6% and 8.4% in the raw ashes to 62.8% and 47.3% in FA1.Ft and FA1.UFt, respectively (Table 2). This is accompanied by a strong decrease in amorphous phase content from 55.5% and 56.7% to 5.3% and 28.6% at 950 °C, respectively. It can be concluded that recrystallisation was the most pronounced in the fine FA1.F fraction, probably due to its fineness and composition which benefits those processes. However, the general content of anorthite and mullite increases in all the fractions; mullite was initially detected in significant quantities only in the coarse FA1.C fraction, and after thermal treatment, it was also detected in the FA1.Mt fraction in the amount of 7.9%, while its content in FA1.C increased from 7.5% to 21.8% after treatment at 950 °C. The quartz content remains stable, with only a slight increase in the coarser fractions, probably related to the overall decrease in sample mass.

Regardless, for the FA2 ashes, the quartz and mullite content remained relatively unchanged, similar to an amount of amorphous phase, indicating the high thermal stability of the FA2 ashes. The amorphous phase content for the FA2.UF fraction decreases only by a few percent from 73.3% to 69.0%, while for the coarser FA2.C fractions, it states 63.7% to 44.3% (Table 3), suggesting that recrystallisation was more likely to occur in larger grains, while smaller grains were more prone to stay in the verified phase or start melting without further recrystallisation. Probably, the cooling condition could influence the recrystallisation process, where a slow cooling rate would promote crystal formation and growth, while fast cooling will “shock freeze” the molten substance that remains in the vitrified phase [93,131,132,133]. Fine fractions as a result of their lower mass and thermal capacity would cool down faster while remaining in the vitrified phase.

Further heating, above 1100 °C, causes decomposition reactions accompanied by exhausted gas release. For all the tested ashes, SO_2_ release was detected in this region, and is primarily attributed to the decomposition of sulphur-containing minerals, in particular calcium sulphate (CaSO_4_) in the form of anhydrite [134,135]. Anhydrite undergoes thermal decomposition typically at temperatures above 1100 °C according to the following Equation (1):

CaSO_4_ (s) → CaO (s) + SO_2_ (g) + 1/2 O_2_ (g) (1)

Such a reaction results in intensive SO_2_ gas release (*m*/*z* 64), contributing to the observed mass loss and the corresponding peak in the DSC curves. The high-Ca FA1 ashes exhibit a more pronounced SO_2_ release compared to the high-Si FA2 ashes. A higher sulphur content is usually found in lignite coals [2,107], and the mineral analysis of the FA1 ashes reveals the presence of significant amounts of anhydrite, reaching 18.8% for the FA1.Ft fraction at 950 °C. Similarly to gehlenite-eckermannite, anhydrite recrystallises from well-dispersed calcium aluminates presented in an amorphous phase abundant in the finer fractions. For the FA1 finer fractions ashes, desulphurisation reactions have the largest share in the total mass loss and state around 11% and 12% for the FA1.UF and FA1.F fractions, while for FA1.M and FA1.C it is only 2.5% and 0.75%. On the contrary, the FA2 ashes have a lower sulphur content and no anhydrite presence was detected. Consequently, a less prominent SO_2_ release and related mass loss can be observed, stating around 1%, 0.8%, 0.3%, and 0.4% for FA2.UF, FA2.F, FA2.M, and FA2.C, respectively, at temperatures above 950 °C.

However, in conjunction with the tendency for sulphur-containing grains to concentrate in the finer fractions, individual differences in SO_2_ release characteristics vary depending on grain size [71,125,136,137]. The small size of the fine and ultrafine particles enhances reactivity and thermal decomposition, leading to more extensive SO_2_ release at lower temperatures. The temperature of the beginning of the individual reactions decreases together with the grain fineness which can be observed for both ash types. For the FA2 ashes, as fineness decreases, the peak of the SO_2_ release appears at lower temperatures, similar to the onset of melting, indicating a higher level of the thermal reactivity of the finer particles. In work [26], the authors performed a thermal analysis of coal fly ash in air and helium atmosphere and stated that around 1000 °C, the source of SO_2_ emissions may be related to the decomposition of sulphur trioxide that is incorporated into the glass phase in the ashes. During heating, these sulphur contaminations undergo decomposition, which is easier to achieve for finer fractions.

SO_2_ emissions from fly ashes can also be influenced by the presence of other minerals and their interactions during thermal treatment. The presence of calcium-rich compounds, such as calcium oxide (CaO) or calcium hydroxide (Ca(OH)_2_), can affect the decomposition of anhydrite [134,135,138]. These compounds can react with the released SO_2_ to form new sulphur-containing phases, such as calcium sulphite (CaSO_3_) or calcium sulphate (CaSO_4_), depending on oxidation conditions [138,139,140], further altering the temperature at which SO_2_ is released and the overall extent of SO_2_ emission.

Some other reactions can take place at elevated temperatures above 1100 °C. The decomposition of calcium-containing minerals and their reaction with the silica (SiO_2_) and alumina (Al_2_O_3_) present in fly ash can result in the formation of dicalcium silicate (Ca_2_SiO_4_), tricalcium silicate (Ca_3_SiO_5_), and tricalcium aluminate (Ca_3_Al_2_O_6_) phases at around 1400 °C [141,142,143]. These phases are crucial when it comes to the use of fly ash in cement production, as calcium silicates and aluminates are fundamental components of cement clinker, contributing to the development of mechanical strength after mixing with water [40,144,145,146,147].

The literature also describes the volatilisation of heavy metals such as mercury (Hg), lead (Pb), and cadmium (Cd) from fly ashes under thermal treatment [76,148,149,150], pointing to their increased content in high-rank coals with enrichment in finer fractions. Together with alkali metals such as sodium (Na) and potassium (K), which also tend to concentrate in finer fractions, volatilisation can lead to hazardous emissions in flue gases, requiring additional gas-cleaning measures to mitigate the environmental impact [151,152,153]. Additional studies should be performed to determine the tendency of heavy metal distribution in the samples tested in this study. The results of chemical analysis only suggest that Pb tends to concentrate in finer fractions, 630 ppm and 466 ppm for the FA1.UF and FA2.UF fractions, which are at similar levels in accordance with the studies [76,148,149,153]. For the coarser fractions, the appearance of Pb was not detected, pointing to its absence or marginal presence below the threshold of detection. The detection of Hg and Cd emissions was not performed in this study.

The differences discussed in thermal behaviour, transformation tendencies, and gas emission characteristics between the FA1 and FA2 ashes and their fractions should be taken into account while determining the type of ash used for potential applications where thermal treatment is used during the production stage and during the exploitation of the final product. Properly chosen type and fly ash fraction can shape the properties of the final product, influencing thermal stability, reactivity, porosity, density, and surface area.

## 4. Potential Applications and Implications

The insights gained from the thermal analysis studies performed on the FA1 and FA2 ashes, particularly the influence of particle size fractionation on their thermal properties, can suggest the best suitable CFA utilisation direction to gain the desired properties in specific applications. This section discusses the potential for the application of the FA1 and FA2 ashes based on the obtained characteristics with attention to the observed thermal events and transformations.

The coarse fraction of the FA1 ash (FA1.C) is characterised by high thermal stability and contains a significant amount of crystalline phases, such as mullite and quartz, making it suitable for use in heat-resistant refractory materials, such as insulating bricks and ceramic filters [154,155,156,157]. Mullite is known for its excellent heat resistance with high melting temperature and can improve the performance and durability of refractories under extreme temperature conditions [158,159]. The controlled thermal treatment of products with FA1.C ash additives can allow high levels of crystallinity to be obtained due to the recrystallisation of the amorphous phase, enhancing thermal stability and resistance to deformation at elevated temperatures of the final product [159,160,161].

The fine fraction of the FA1 ashes (FA1.F) demonstrates the highest level of the thermal recrystallisation of the glass phase, resulting in the amorphous phase recrystallisation of around 90% at 950 °C. This property may be desirable during the production of clinker, where the presence of high-temperature fine aggregates that recrystallise in aluminosilicate phases is beneficial [39,40,146,147]. The recrystallisation of FA1.F into anhydrite can be used in the production of special cements, such as calcium aluminate cement, sulfoaluminate cement, and belite-rich cement [143,144,145,162,163,164,165,166].

For the FA2 ashes, the early onset of melting and agglomeration observed in the fine (FA2.F) and ultrafine (FA2.UF) fractions may limit their use as a substitute in cement clinker production processes [167,168]. The presence of alkaline glass structure modifiers can lead to the formation of low melting point phases, which can negatively affect the production process by inducing slagging and early agglomeration [169,170], and as described in [171], to meet the desired clinker quality, individual thermally induced reactions should take place in controlled time at the respective temperature stages of the kiln.

However, characteristics such as high fineness and the contamination of a significant amount of reactive alumina and silica make FA2.UF a fine fraction and a promising pozzolanic additive for high-performance concrete [172,173,174]. The addition of such pozzolanic materials increases the density of the concrete matrix, improves strength and durability, and increases resistance to chemical attack [175,176,177,178]. In addition, the high thermal stability of these ashes can be desired in special concrete applications exposed to elevated temperatures, such as chimneys, industrial floors, and thermal energy storage systems [179,180,181,182,183].

The low melting point of the FA2.UF and FA2.F fractions and low levels of gas emission make them suitable for use in areas such as ceramic production [184,185,186]. The formation of a liquid phase at relatively low temperatures around 950 °C can be used to promote the melting and sintering of ceramic glaze, while the fineness of the particle size ensures a smooth and homogeneous surface [187,188,189,190]. The FA2.M fraction, due to its high spherical grain content and high thermal stability, allows its use as filler or additive in high-temperature ceramic applications without the risk of thermal deformations and cracks in the material during production and later during exploitation even under harmful conditions (chemically aggressive environment, exposure to extremal temperatures, or harsh mechanical conditions) [184,187,191]. Notable is a spinel appearance for the FA2.F and FA2.UF fractions after the thermal treatment, which can be beneficial in special spinel refractory materials, and as was reported by Nguyen et al. [129], such an additive can improve both the modulus of rupture and the resistance to thermal shock, while the impact on refractory properties is minimal.

The coarse fraction of the FA2 ashes (FA2.C) with its large porous grains contains the highest amount of unburned carbon, making it a promising candidate for use in thermal insulation materials and lightweight aggregates. As observed in the current study, the heating of the coarse FA2 fraction at around 650 °C results in the extensive volatilisation of carbon contaminations. If trapped, such gases will create a high level of porosity within the particles, resulting in an increase in the porosity of the material and a decrease in density [25,110,192,193,194]. Thermal stability at higher temperatures ensures its performance and durability as a high-temperature insulation additive.

The medium fraction of the FA1 ashes (FA1.M) has a combination of abrasive particles (as quartz) and a glassy phase rich in aluminium-silicate and calcium-silicate compounds. These characteristics make FA1.M a potential filler material in high-temperature friction materials such as brake pads [195,196,197]. The presence of hard abrasive particles in composites prepared with fly ash additive can enhance the friction properties and wear resistance, while the glassy phase can act as a high-temperature binder, improving the mechanical strength and thermal stability during exploitation. The thermal stability of fly ashes ensures their performance and integrity under high-temperature exploitation conditions, which in addition can potentially reduce the reliance on traditional materials, such as asbestos, and provide a sustainable and eco-friendly alternative [198,199].

Regardless, the fly ash fractions obtained still exhibit a wide range of properties due to varying compositions, such as Fe-bearing compounds, unburned coal, and mineral and glass grains in different proportions. These variations can significantly influence both the production processes and the characteristics of the final product. Further processing and treatment of the obtained fractions can yield exceptional materials with highly desirable properties for specific applications. Although this work focused on granulometric classification and thermal treatment, the potential for additional separation techniques, such as magnetic and electrostatic field separators, offers promising avenues for future research. These methods could lead to more refined ash separation pathways and targeted applications.

In addition, the methodology developed in this study can be extended to other industrial wastes, including slags, bottom ashes, and landfilled ashes. This broader application aligns with the Best Available Technology (BAT) principles and advances material engineering traditions.

These examples do not exhaust the topic but only demonstrate how the thermal properties and characteristics of different fly ashes and their fractions can be leveraged to develop tailored solutions for various applications. The knowledge and understanding of thermal behaviour can help engineers and material scientists optimise the performance, durability, and sustainability of final products. The development of new ways to use fly ash not only brings benefits to the properties of the final product and creates environmentally friendly materials but also provides new market areas for the utilisation of industrial by-products, driving innovation, and reducing the amount of fly ash that goes into landfill.

## 5. Conclusions

This study investigated the thermal behaviour of FA1 calceus and FA2 silicious fly ashes and their granulometric fractions employing such techniques as thermogravimetry (TG), differential scanning calorimetry (DSC), and evolved gas analysis (EGA). The distinct differences in the thermal behaviours were revealed and discussed with attribution to the variations in chemical composition, mineralogy, and particle size distribution, influenced by the rank of the parent coal and combustion conditions. Thermal analysis has identified distinct temperature regimes, each associated with specific processes and transformations, such as the evaporation of physically bound water; the dihydroxylation, decarbonation, oxidation, and melting of aluminosilicate glasses; and the decomposition of sulphur-bearing minerals.

The thermal properties were significantly impacted by the type of granulometric fraction. The finer fractions exhibit enhanced reactivity, lower combustion temperatures of organic residuals, and a higher tendency toward thermoplasticity, especially for finer fractions. The low-rank coal ashes (FA1) show higher thermal stability and lower thermally induced meltability compared to the high-rank coal FA2 ashes. However, the recrystallisation of the amorphous phase during thermal treatment is more pronounced in the FA1 ashes, particularly in the finer fractions, resulting in the formation of crystalline phases such as gehlenite-akermatine and anhydrite.

The study highlights the importance of thermal analysis in characterising fly ash fractions and understanding their potential for high-temperature applications. The aerodynamic separation of fly ashes into different size fractions yielded materials with varied thermal properties and reactivity, suitable for specific industrial uses. This approach demonstrates the potential for developing customised solutions for fly ash utilisation, which contributes to more efficient resource management and environmentally friendly material development.

The findings provide a foundation for further research on advanced separation techniques and the in-depth characterisation of fly ash fractions. This work contributes to the development of comprehensive recycling systems and material characterisation methods, which could expand beyond fly ash to other industrial wastes. Such studies are crucial for optimising resource utilisation and promoting sustainable materials engineering practices, aligning with the Best Available Technology principles, and advancing the field of materials science.

## Figures and Tables

**Table 1:** Chemical composition of FA1 and FA2 fly ash fractions.

[%]	FA1.O	FA1.C	FA1.M	FA1.F	FA1.UF	FA2.O	FA2.C	FA2.M	FA2.F	FA2.UF
SiO_2_	30.9	44.4	28.8	21.1	16.2	54.2	58.9	51.3	49.7	48.0
Al_2_O_3_	30.1	33.1	28.8	26.0	24.1	24.5	23.7	23.0	29.5	30.1
CaO	24.7	12.5	25.4	33.9	37.8	5.1	3.0	6.6	4.3	4.2
SO_3_	0.2	2.1	3.4	4.7	6.4	0.4	0.3	0.4	1.0	1.3
Fe_2_O_3_	9.5	5.7	10.7	10.6	10.4	8.3	7.0	9.1	6.1	5.9
MgO	1.1	0.8	1.0	1.3	1.5	3.4	2.5	4.2	2.9	2.5
TiO_2_	0.7	0.9	0.7	0.6	0.6	1.0	0.9	0.9	1.3	1.3
P_2_O_5_	0.6	0.4	0.6	0.5	0.6	0.3	0.2	0.3	0.7	0.9
K_2_O	0.2	0.2	0.1	0.1	0.2	2.6	2.7	2.6	3.3	3.2
Na_2_O	-	-	-	-	-	1.0	0.6	1.5	2.3	2.4

**Table 2:** Mineralogical composition of FA1 fly ash fractions.

	Untreated					Treated at 950 °C				
[%]	FA1.O	FA1.C	FA1.M	FA1.F	FA1.UF	FA1.O.t	FA1.C.t	FA1.M.t	FA1.F.t	FA1.UF.t
Gehlenite	10.1	2.2	13.2	17.6	8.4	23.2	7.0	24.8	62.8	47.3
Quartz	2.5	6.8	1.3	-	-	2.7	9.9	0.8	-	-
Hematite	6.7	-	3.6	5.1	5.3	11.8	5.1	12.3	9.2	7.4
Mullite	-	7.5	-	-	-	14.3	21.8	9.7	-	-
Anorthite	5.0	4.5	6.5	-	-	9.5	14.1	10.2	0.8	0.6
Anhydrite	4.5	-	-	-	15.8	5.5	-	5.1	18.8	14.8
Cristobalite	-	-	14.1	-	-	1.6	1.5	-	-	-
Lime	-	-	-	-	2.1	-	-	-	-	-
CCaA ^1^	1.3	0.0	8.7	20.8	8.4	7.0	10.3	-	-	-
Amorphous	69.7	78.8	52.3	55.5	56.7	27.7	33.2	35.0	5.3	28.6

^1^ complex calcium aluminates, marked with a star in Figure 2.

**Table 3:** Mineralogical composition of FA2 fly ash fractions.

	Untreated					Treated at 950 °C				
[%]	FA2.O	FA2.C	FA2.M	FA2.F	FA2.UF	FA2.O.t	FA2.C.t	FA2.M.t	FA2.F.t	FA2.UF.t
Mullite	20.2	14.1	22.2	18.1	19.7	27.6	25.2	21.9	21.2	22.4
Quartz low	17.2	20.3	15.2	6.0	5.9	17.4	28.5	14.5	4.9	5.3
Hematite	1.4	1.3	2.6	0.7	0.8	6.9	5.7	10.1	2.5	2.2
Lime	0.3	0.5	1.2	-	-	0.4	0.2	0.7	-	-
Spinel	-	-	-	-	-	-	-	-	4.4	4.2
Periclase	-	-	-	-	-	1.5	0.8	2.0	-	-
Anatase	-	-	-	-	-	0.5	0.3	1.1	0.5	-
Amorphous	62.1	63.7	58.5	74.4	73.3	50.7	44.3	54.7	69.2	69.0

**Table 4:** Total loss of ignition (LOI wt%) at 950 °C of FA1 and FA2 fly ash fractions.

**FA1.O**	**FA1.C**	**FA1.M**	**FA1.F**	**FA1.UF**	**FA2.O**	**FA2.C**	**FA2.M**	**FA2.F**	**FA2.UF**
1.7	6.5	0.9	1.7	1.8	11.2	14.9	7.7	13.3	13.3

## Data Availability

The data used to support the findings of this study are included within the article.
